# Ultrastructural studies of Intraflagellar Transport trains in Chlamydomonas reinhardtii suggest a revision of the current model for IFT trafficking in the flagellar compartment

**DOI:** 10.1186/2046-2530-4-S1-O16

**Published:** 2015-07-13

**Authors:** E Vannuccini, E Paccagnini, F Cantele, M Gentile, D Dini, F Fino, C Mencarelli, Pietro Lupetti

**Affiliations:** 1 Dept. of Life Sciences, University of Siena, Siena, Italy; 2 Dipartimento di Chimica Università di Milano, Milano, Italy

## 

Intraflagellar Transport (IFT) is the molecular process responsible for the active bidirectional trafficking of structural and functional components that occurs in the flagellar compartment of eukaryotic cells. Flagellar components undergo a constant turnover at flagellar tip and multiple evidences indicate that flagellar elongation, maintenance and reabsorption depend on the correct balance between anterograde and retrograde trafficking. IFT particles are formed by >22 polypeptides assembled into two subcomplexes, A and B, and are moved bidirectionally along the outer surface of axonemal doublets as linear rows of IFT particles, for which we proposed the term "train". Anterograde IFT trains are moved by kinesin II and carry to flagellar tip the retrograde motor cytoplasmic dynein 1b, responsible for retrograde IFT. In a previous study carried out on *Chlamydomonas *flagella we identified two types of IFT trains we named long and short trains, each characterized by a specific ultrastructure and a definite internal repeat, and proposed that long, less compact trains could represent anterograde IFT while the short, more compact trains could be retrograde. To challenge such model, we monitored by transmission electron microscopy the IFT trains expressed both in wt regenerating flagella and during flagellar reabsorption induced in the ts mutant pf1-fla10. We also progressed in our electron tomographic 3D modeling of short IFT trains. Our data suggest that long IFT trains are not the only anterograde IFT component. Rather, anterograde IFT is contributed also by a subclass of short trains that is expressed in a flagellar length-dependent fashion.

